# Antifungal Activity of Conjugated Metal Organic Frameworks: A Multidisciplinary Undergraduate Laboratory Experiment

**DOI:** 10.1021/acs.jchemed.5c01733

**Published:** 2026-05-19

**Authors:** Tina Skorjanc, Safa Gaber, Martina Bergant Marušič, Dinesh Shetty, Matjaz Valant

**Affiliations:** † Materials Research Laboratory, 119110 University of Nova Gorica, Vipavska 11c, 5270 Ajdovscina, Slovenia; ‡ Department of Chemistry and Center for Catalysis & Separations (CeCaS), 105955 Khalifa University of Science & Technology, P.O. Box 127788, Abu Dhabi 127788, United Arab Emirates; § Laboratory for Environmental and Life Sciences, 119110 University of Nova Gorica, Vipavska 13, 5000 Nova Gorica, Slovenia

**Keywords:** Upper-Division Undergraduate, Interdisciplinary/Multidisciplinary, Hands-On Learning/Manipulatives, Materials Science, Microbiology, Applications of Chemistry, Antimicrobial Activity, Students

## Abstract

Mechanochemical synthesis, purification, and basic characterization of a Cu­(II)-based MOF are described as an experiment for upper-level undergraduate students. The chemistry/materials component of the laboratory course is combined with a microbiology module into a two-session multidisciplinary student activity. Students are divided into groups as they investigate the antimicrobial activity of the prepared MOF by three different assays, namely, O.D. measurements, Presto Blue, and Crystal Violet assays, using*Candida albicans*as a safe and well-understood model organism. This approach to the microbiology module encourages student collaboration, data sharing, and critical thinking during the result interpretation. Through this exercise, students learn about the value of different approaches in tackling a specific scientific problem, i.e., the antifungal activity of a MOF. Using OD measurement as an example, they also learn to critically assess whether a well-established scientific protocol can be adapted to specific contexts.

## Graphical abstract

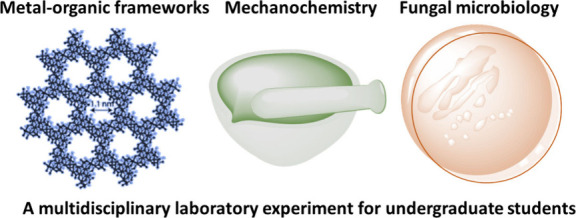

## Introduction

Porous materials, including porous organic polymers (POPs), metal–organic frameworks (MOFs) and covalent organic frameworks (COFs), are emerging classes of materials characterized by extensive surface areas which can extend to several thousand square meters per gram of material. Large surface areas enable the formation of interactions with various small molecules and tunability of their structures enables the design of materials suited to a range of needs. These materials have thus found uses in various applications ranging from gas capture and separation,^1^ catalysis,^2^ sensing,^3^ pollutant removal,^4^ to biomedical applications, such as drug delivery,^5^ bioimaging,^6^ and antimicrobial materials.^7^ Several features of these materials make them suitable for biomedical applications, including the following:^8^

(1) **Structural tunability** enables the introduction of chemical functionalities tailored to a specific application, the designability of pore sizes and volumes, and the predictability of the physical properties such as polarity, hydrophobicity, and surface chemistry. Postsynthetic modifications, for instance, conjugation of targeting or other molecules, metalation, or formation of hybrid materials, provide an additional avenue for adapting the structure when desired.
(2) **Stimulus responsiveness** can be rationally introduced when desired. For example, some materials have shown pH-dependent cargo release, sensitivity to a competitive binder, light sensitivity, or redox-dependent behavior. These properties have been utilized, for example, for on-demand drug release.
(3) **Porosity** enables the encapsulation of larger amounts of therapeutic cargo within the carrier compared to traditional nonporous delivery systems such as liposomes, micelles, and organic or inorganic nanoparticles. Enhanced drug-loading capacity not only reduces the frequency of administration but also helps minimize treatment-related side effects, improving the overall efficacy and patient compliance.
(4) **Biocompatibility** of POPs and COFs tends to be high because of their purely organic nature. In this regard, COFs are often described as superior to the related metal–organic frameworks (MOFs). Their biocompatibility can be further improved when the particles are functionalized with biocompatible groups, such as polyethylene glycol (PEG).
(5) **Biodegradability**. The use of reversible dynamic covalent bonding and/or coordination bonding in the synthesis of these materials enhances their potential for biodegradability, offering an advantage over traditional polymeric materials. Many of the linkages employed in the preparation are responsive to external stimuli such as pH, which further enhances their long-term biodegradability potential. This responsiveness not only facilitates controlled degradation but also promotes the environmental sustainability over time.

Although these classes of materials have found their place in recent undergraduate student textbooks as emerging materials, it is rare that they are included in college-level laboratory courses. With a 2025 Nobel Prize in Chemistry recently being awarded for the discovery of MOFs, it is expected that the interest in these materials will increase among university students and the general public in the coming years. While it may be difficult to plan a student laboratory exercise that incorporates MOF preparation, characterization, and biomedical application in a short time frame, testing the antifungal activity of MOFs that can be formed in relatively short reaction times is a reasonable experimental activity that can be completed over the course of two laboratory sessions. During the first laboratory session, students synthesize Cu­(II)-based MOFs by following a detailed experimental procedure given in the lab manual, purify and dry them. CuTp MOF, composed of Cu^2+^ metal ions and 1,3,5-triformylphloroglucinol (Tp) linkers, is proposed as a viable example because its preparation involves mechanochemistry, a simple yet often overlooked method of preparation. Furthermore, the synthesis of this MOF is accomplished in 4 h, which is a reasonable reaction time for a student laboratory experiment. In the second session, students characterize the prepared MOF samples with Fourier-transform infrared (FT-IR) spectroscopy and powder X-ray diffraction (PXRD). In the same session, they also investigate the antifungal activity of the prepared and purified materials by various methods: a proliferation assay that measures optical density (O.D.), a bioactivity assay based on the Presto Blue reagent, and a biofilm inhibition assay with Crystal Violet. The main goal of this laboratory exercise is not to elucidate the mechanism of antifungal activity of CuTp MOFs but rather to train students to evaluate bioassay data and assay suitability.

While some interdisciplinary student experiments at the interface of chemistry and microbiology have previously been proposed for a teaching laboratory, these are not in the context of porous materials. Rather, these experiments focus on antimicrobial peptides,^9^ silver-cellulose composite materials,^10^ or small organic molecules.^11^ Furthermore, their focus is not on comparing the various antimicrobial assays, as they focus on a single assay. Some student activities related to MOFs have been reported, but they primarily deal with the structure of MOFs^12,13^ or environmental applications.^14,15^ We therefore consider the novelty of the current student laboratory exercise in both the type of application of MOFs and the breadth at which this application is explored in a student setting.

## Experiment Overview

### Chemistry

Upper-level undergraduate students may work either individually or in groups of two students during a 6 h laboratory session. Students compare and discuss their results at the end of the class.

During the first session, the students synthesize the target MOFs by reacting the metal salts (CuCl_2_·2H_2_O) with commercially available organic linker, Tp. Depending on their level, they may be asked to follow a synthetic procedure directly from the literature,^16,17^ or they may be given a handout included in the Supporting Information of this manuscript. The synthesis is based on mechanochemistry, where 0.15 mmol of Tp, 0.225 mmol of CuCl_2_·2H_2_O and 200 μL of water are ground with a mortar and pestle in the absence of any organic solvent or catalyst. When a uniform mixture is obtained, the contents are reacted in an oven at 90 °C for 4 h. Finally, the powder product is washed with several solvents (DMA, THF, water, acetone) and dried in a vacuum dryer. All experimental procedures should be carried out independently by the students, including reagent weighing, mechanical grinding, thermal treatment, washing and drying.

If relevant instrumentation is available at your institution, the FT-IR spectrum and the PXRD pattern of the synthesized material are measured and compared to the literature to confirm the chemical functionality and crystallinity, respectively. The operation of these instruments requires instructor supervision for setup and safe operation. However, students should be actively involved in these measurements by preparing samples, loading samples into instruments, selecting and applying predefined measurement parameters, and analyzing the measured data. Instructors should not conduct experiments on behalf of students but rather provide supervision and technical guidance as needed. Interpretation of FT-IR peaks is typically part of organic chemistry classes in the second year of undergraduate programs, whereas PXRD pattern analysis is subject to more advanced, elective classes in materials science, so this part can be adapted to your students. Conjugated CuTp MOF has been synthesized and reported several times, so its synthesis can be considered standardized. Even so, recording FT-IR and PXRD data is valuable and strongly recommended whenever possible; it may be omitted only because of instrumentation constraints.

### Microbiology

The antifungal activity of the synthesized MOFs was evaluated with*C. albicans* as a model organism using three different antimicrobial tests. The model organism is chosen because it is widely available, falls in the biosafety level 1 (BSL 1), so it is safe to use in a student laboratory, and it is easy to culture. Approximately 2 h should be budgeted for the first part of the microbiology experiments, and another 2 h are needed to perform the assays on the following day.

In this laboratory session, students should be divided into groups, so that each group performs one type of antifungal test, namely, the optical density measurements, the Presto Blue assay, or the Crystal Violet assay. Then, the student groups share the results with each other in order to gain a deeper understanding of the types of information that can be gathered from every antifungal test.

All students start the microbiology experiments in the same manner by preparing a stock suspension of*C. albicans* and incubating the fungal cells with different concentrations of the MOFs in 48-well plates (details of the experiment given in the Supporting Information). After overnight incubation, each group follows a protocol for a different assay. The O.D. method measures turbidity, so it is indicative of the number of fungal cells, but it is unable to distinguish between the metabolically viable and nonviable cells. The fluorescence-based Presto Blue assay, in contrast, considers metabolically active cells by measuring the activity of a reductase enzyme that reduces resazurin to a fluorescence product called resorufin. Finally, the Crystal Violet assay quantifies the extent to which the MOF inhibits the formation of*C. albicans*biofilms.

The use of the microplate reader requires instructor assistance in setting up the relevant method, but all aspects of the microbiology experiment are carried out hands-on by the students. Students independently prepare serial dilutions of MOF suspensions, fungal cultures to be used in antifungal tests, set up all antifungal assays, and perform data analysis, plot relevant graphs, and interpret the results. They also operate the preset microplate reader methods by themselves. As in the chemistry portion of the laboratory, instructors are intended to supervise students and not perform experiments on their behalf.

## Student Learning Outcomes

The laboratory experiment was developed around learning outcomes integrating chemistry, microbiology, and data interpretation with undergraduate students at the University of Nova Gorica, Slovenia. The details of the learning outcomes and the laboratory activities supporting them are summarized in Table 1. While first-year students were able to follow instructions and carry out the exercise, they lacked some of the background in spectroscopy; therefore, the laboratory experiment herein described is recommended for upper-level undergraduate students.

**Table 1:** Student Learning Outcomes, Associated Laboratory Activities and Assessments

Learning outcome	Laboratory activity	Evidence of learning outcome attainment
Performing mechanochemical synthesis of a MOF and its purification	Weighing chemicals, mechanochemical grinding, thermal treatment, washing with solvents	Laboratory notebook entries, product yield, product color, lab report
FT-IR spectroscopy and PXRD measurements	Sample preparation for FT-IR and PXRD, comparison of obtained data with reference spectra	Correct assignment of FT-IR peaks, identification of PXRD reflections, lab report
Conducting microbiological experiments	Preparation of*C. albicans*suspensions, serial dilution of MOFs, microplate setup, work in sterile conditions	Absence of contamination, dose-dependent effect of MOF on the growth of fungi
Execution of various antifungal assays	Optical density measurements, Presto Blue assay, Crystal Violet assay	Plots with correctly labeled axes for each assay, lab report
Critical evaluation of each antifungal assay	Comparison of assay results among different student groups, discussion on MOF absorbance	Lab reports, answers to student discussion questions
Development of collaborative and problem-solving skills	Group-based microbiology experiments, data sharing among groups	Group discussions, answers to student discussion questions, interpretation of shared data

## Hazards

Gloves, lab coats, and safety goggles were used throughout the experiment. All manipulations were carried out in a chemical fume hood (MOF synthesis and washing) or in a biosafety cabinet (microbiology experiments).

Several chemicals used in the preparation and purification of the MOFs as well as Crystal Violet used in the microbiology session pose a health risk and should therefore be handled with care. The details of specific hazards are given in the Supporting Information under the Hazards and Safety section of both MOF synthesis and microbiology sections.

*Candida albicans* (ATCC 10231) is a yeast that is classified as BSL-1 and requires a basic level of containment that relies on standard microbiological practices with no special primary or secondary barriers recommended other than a sink for hand washing.

## Results and Discussion

The synthesis of the CuTp MOF involves mechanical grinding of CuCl_2_·2H_2_O and 1,3,5-triformylphloroglucinol (Tp) with a drop of water until a uniform mixture is obtained followed by 4 h of heating at 90 °C (details in the Supporting Information). This relatively short reaction time allows for the implementation of this MOF in a student laboratory. The purification procedure is also simple and involves only washing with N,N′-dimethylacetamide (DMA), tetrahydrofuran (THF), water and acetone. Washing is carried out by adding the above solvent to the MOF powder in a centrifuge tube, shaking the tube vigorously, centrifuging the suspension, and discarding the supernatant every time. Finally, the samples are dried in a vacuum dryer overnight or on air for a longer time period.

It is highly recommended that students perform some basic characterizations of the prepared MOF samples at the beginning of the second session. FT-IR spectra and PXRD patterns of CuTp are shown in Figure 1 (collected by students with the aid of an instructor) with the intention that these data serve as a reference for the students. These two characterizations will confirm the molecular-level structure and crystallinity of the synthesized materials, respectively.

**Figure 1:**
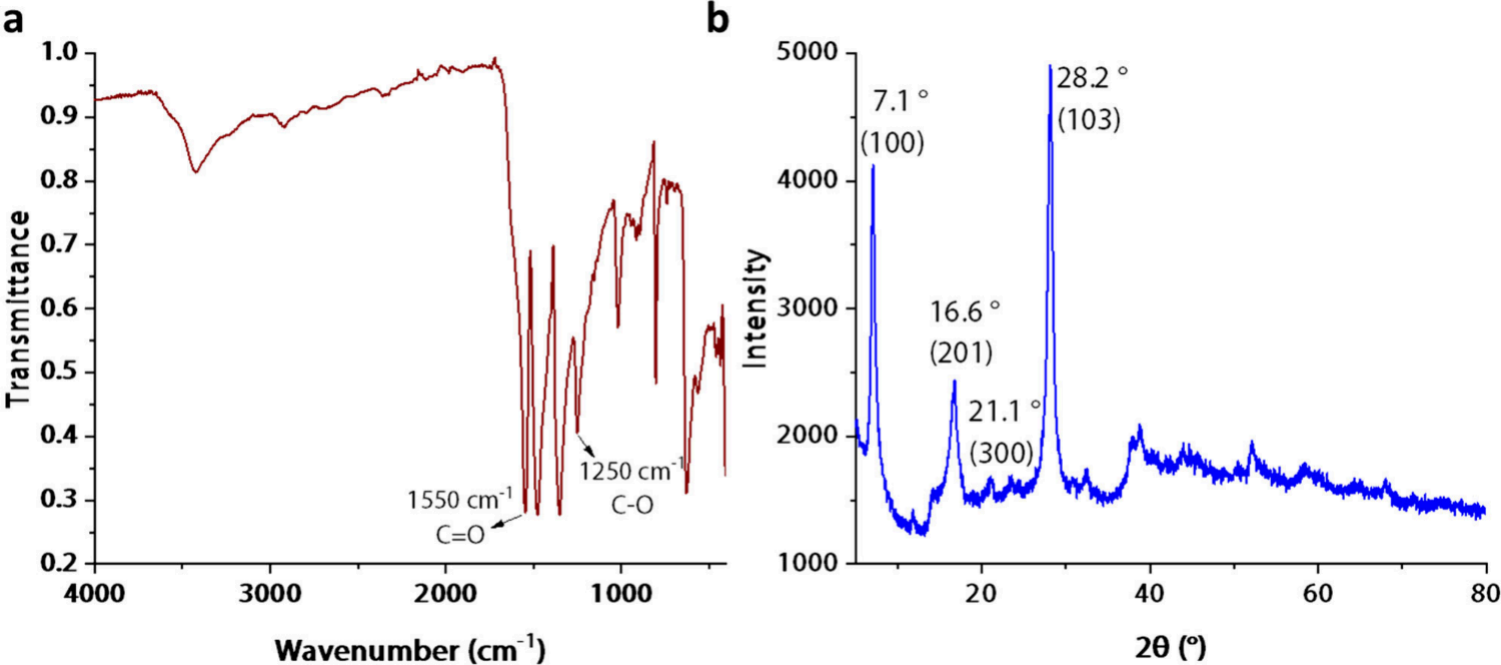
Basic characterization of CuTp MOF that should be done at the beginning of the second session: (a) a reference FT-IR spectrum of CuTp; (b) a reference PXRD pattern of CuTp MOF. These spectra were recorded as a part of the student laboratory course and they match the data in ref 16.

The microbiology experiments start in the second laboratory session and in the first part they are essentially the same regardless of the assay that will be performed. Students are given an overnight culture of*C. albicans* with a known value of O.D., which they dilute in the Yeast-Peptone-Dextrose (YPD) medium to an O.D. of 0.1, followed by another 1:50 dilution in YPD. Next, students prepare MOF suspensions. For better dispersion, it is recommended to first disperse the MOF particles in DMSO (a total of 5% v/v in the final suspension) and then add the YPD broth. As shown in Figure 2, stock solutions of MOFs are prepared in such a way that the final concentrations in the wells range between 1.00 and 0.031 mg/mL obtained in a serial dilution. As each well gets 100 μL of the MOF suspension and 150 μL of the*C. albicans* suspension, the prepared MOF stock solutions should range in concentration between 2.50 and 0.078 mg/mL. The control wells should have 100 μL of YPD rather than a MOF suspension. Next, students add 150 μL of the 1:50 dilution of*C. albicans*to the wells in columns with the MOF samples (1, 2, and 3) and the control. The blank contains 250 μL of the YPD broth. The plates are incubated in a static incubator at 37 °C overnight.

**Figure 2:**
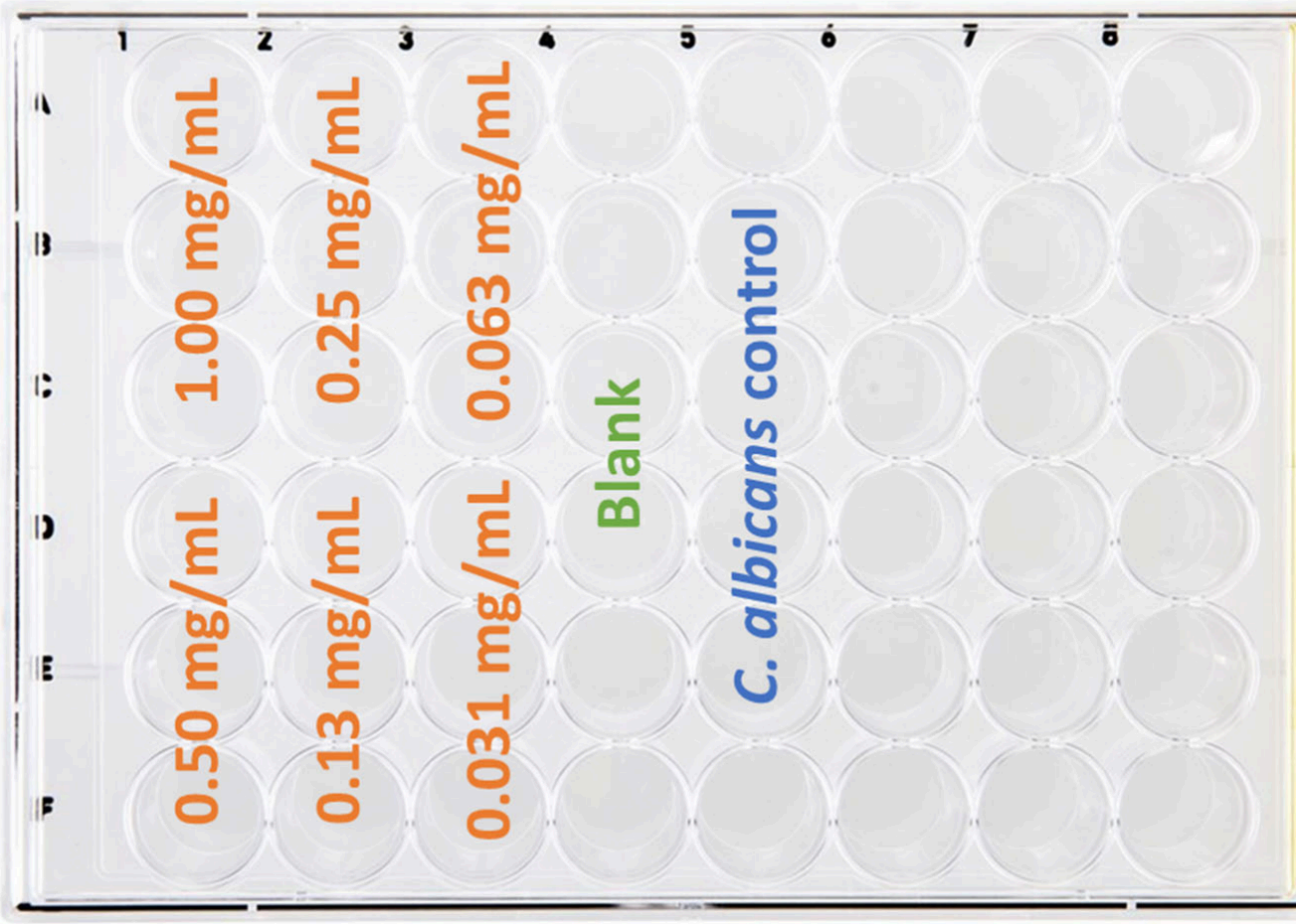
An example of the setup for all three types of antifungal assays in a 48-well plate. Columns 1, 2, and 3 are used for different concentrations of MOFs, so that every concentration is measured in triplicate. Every well contains*C. albicans*in YPD and a particular MOF condition in 5% DMSO–95% YPD. Row 4 serves for the blank (the YPD medium only), and row 5 is for the controls (*C. albicans* + YPD).

After the incubation period is finished, every student group performs a different type of antifungal test. The fastest to perform are the O.D. measurements in a microplate reader. The instructor is recommended to set up a method to measure the absorbance at 600 nm prior to the class. As many MOF samples, including the CuTp MOF, are colored and absorb light at around 600 nm, this type of assay is not the most suitable for evaluating antifungal activity. At higher MOF concentrations, higher O.D. readings are due to the absorption of the MOF rather than the presence of a large number of fungal cells. By plotting a graph of % yeast growth vs MOF concentration as shown in Figure 3a, students should realize this and recognize the drawbacks of this specific method in the context of CuTp and many other MOFs.

**Figure 3:**
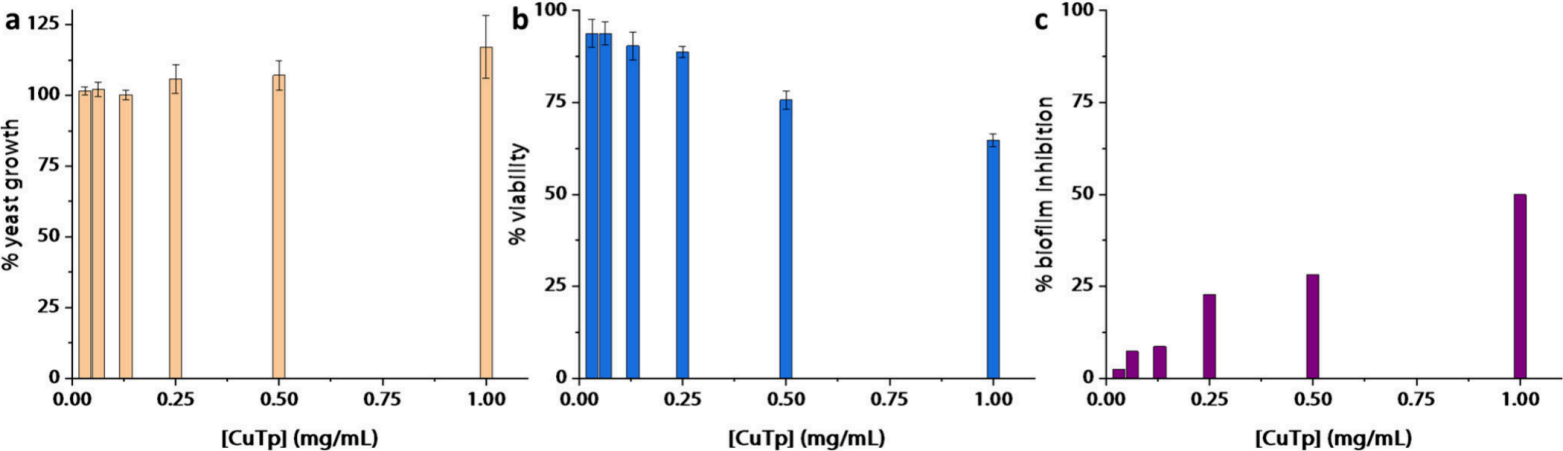
Results of the three antifungal assays (data collected by students under instructor supervision): (a) optical density measurement results; (b) Presto Blue assay results; (c) Crystal Violet assay results.

In the Presto Blue assay, students add 5.0 μL of the commercial reagent into every well and incubate the plate in the dark at 37 °C for 30 min. Next, they measure the fluorescence signal at 590 nm upon excitation at 560 nm in a microplate reader. It is recommended that the instructor sets up a method on the available microplate reader prior to the class. Students can calculate the % viability of*C. albicans*cells by using the formula % viability = (test sample values)/(control values) × 100. Then, a plot of % viability vs MOF concentration can be drawn using these data (Figure 3b).

The assay with Crystal Violet is the most time-consuming of the three, but very valuable because it gives information on the inhibition of fungal biofilms. After the 24-h incubation of*C. albicans*with different concentrations of the MOFs, the nonadherent cells are removed, the biofilms are gently washed with PBS, and then incubated with 0.2% aqueous solution of Crystal Violet for 30 min in the dark. Excess dye is subsequently removed, the biofilms are washed again with PBS, and destained with 33% aqueous solution of acetic acid. The absorbance of destaining solutions is then measured at 590 nm in a microplate reader, and % biofilm inhibition can be calculated. Finally, a plot of % eradication vs MOF concentration can be plotted (Figure 3c).

Figure 3 shows the results of the three assays obtained in a student’s laboratory setting. These data can serve as a reference point for experiments performed by other student groups. It is vital for the students to understand the different information that each plot shows. The O.D. assay shows % yeast growth and has significant drawbacks in the case of compounds that absorb light near 600 nm. The Presto Blue assay shows % viability; therefore, it is expected that higher MOF concentrations will show lower values. In contrast, the Crystal Violet assay shows % eradication, so higher MOF concentrations are expected to show higher values. Furthermore, students should understand how MOF absorbance in the O.D. measurements is responsible for the increased values at higher concentrations of MOF, even the physically illogical % viability of over 100%.

As part of the student discussion, the structure–function relationship between CuTp and its antifungal activity should be clarified. CuTp contains Cu^2+^ ions, which are known for their antimicrobial activity against Gram-negative and Gram-positive bacteria as well as fungi.^18^ The mechanism behind the toxicity of these ions relies on the reduction of Cu^2+^ ions into Cu^+^ ions, which generate reactive oxygen species (ROS) that damage macromolecules, including proteins, DNA, and cell membranes.^19^ Students may ask about the usefulness of a MOF in such an application when simple Cu^2+^ salts can also act as antifungal agents. The answer is not trivial, as it incorporates several factors and is related to the structure of the MOF: (i) copper salts create a burst release of Cu^2+^ ions, which can have serious side effects and is less effective in the long run than the effect of metal ions from a MOF which is more sustained,^20^ (ii) high surface areas of the MOF enhance contact with fungal cells, thereby improving the antifungal activity,^21^ (iii) MOFs often have better biocompatibility than metal salts,^22^ and (iv) the generation of ROS from a MOF is often higher than from metal salts due to the synergistic effects of the metal nodes and organic linkers.^21^ While other Cu-based MOFs could be used for the purpose of this student laboratory exercise, most require longer reaction times and are therefore more difficult to apply in a student setting.^23^

To evaluate whether the learning objectives of the laboratory experiment were met, a questionnaire was designed for students to fill out. This questionnaire has been included in the Supporting Information and can be adapted by instructors according to their needs. The majority of the students evaluated the laboratory exercise positively, and our survey results averages indicate that the learning objectives have mostly been met (page SI-14). Students have struggled the most with the evaluation of PXRD and FT-IR data, which can be attributed to the fact that undergraduate courses cover these experimental techniques, especially X-ray diffraction, only to a limited extent. This was also indicated in the open-ended question section of the survey. In contrast, the majority of students felt that they were able to perform the synthesis and purification of an MOF as well as to conduct different biological assays to evaluate antifungal activity. The vast majority of students felt that they were able to contribute to group work and that they improved their ability to share and interpret data and to solve problems. The laboratory experiment presented herein falls within the scope of standard classroom activities, so no formal ethics review was conducted. No personally identifiable information was collected. No unexpected or unusually high safety hazards were encountered during the experiments.

## Summary

The proposed laboratory activity for undergraduate students is composed of chemistry and microbiology components. In the first part, students learn about the mechanochemical synthesis of a Cu-based MOF and perform some basic characterizations that are typically applied to MOFs, including PXRD and FT-IR spectroscopy measurements. In the second part of the laboratory exercise, students use the synthesized MOF to apply it to microbiological experiments. They use various assays, including optical density measurements, Presto Blue assay, and Crystal Violet assay, to learn about the standard methods of evaluating antimicrobial activity using*Candida albicans*as a model microbe. While elucidating mechanisms of antifungal activity of the MOF is beyond the scope of this student laboratory, critical evaluation of bioassay data and suitability of different bioassays are important learning outcomes of the exercise.

The expected student learning outcomes include (1) gaining experience with mechanochemistry as a method of material preparation, (2) understanding what porous materials and MOFs are, (3) performing some basic materials characterization methods such as PXRD and FT-IR spectroscopy, (4) experimenting with standard microbiology techniques to study antifungal activity, (5) learning to critically evaluate the strengths and weaknesses of each microbiology assay as well as its suitability in the context of MOFs, and (5) appreciating the multidisciplinary nature of modern science.

## Supplementary Material



## Notes

Student handout with an overview of the chemical and microbiological experiments, detailed experimental procedures, questions for student discussion, instructor notes with list of chemicals, equipment, hazard, instrumentation and answers to student discussion questions (DOCX)

## References

[1] Jia T., Gu Y., Li F. (2022). Progress and Potential of Metal-Organic Frameworks
(MOFs) for Gas Storage and Separation: A Review. J. Environ. Chem. Eng..

[2] Felix
Sahayaraj A., Joy Prabu H., Maniraj J., Kannan M., Bharathi M., Diwahar P., Salamon J. (2023). Metal–Organic
Frameworks (MOFs): The next Generation of Materials for Catalysis,
Gas Storage, and Separation. J. Inorg. Organomet.
Polym. Mater..

[3] Kamal S., Khalid M., Khan M. S., Shahid M. (2023). Metal Organic
Frameworks
and Their Composites as Effective Tools for Sensing Environmental
Hazards: An up to Date Tale of Mechanism, Current Trends and Future
Prospects. Coord. Chem. Rev..

[4] Jin Y., Liu H., Feng M., Ma Q., Wang B. (2024). Metal-organic
Frameworks
for Air Pollution Purification and Detection. Adv. Funct. Mater..

[5] Lawson H. D., Walton S. P., Chan C. (2021). Metal–Organic Frameworks for
Drug Delivery: A Design Perspective. ACS Appl.
Mater. Interfaces.

[6] Horcajada P., Chalati T., Serre C., Gillet B., Sebrie C., Baati T., Eubank J. F., Heurtaux D., Clayette P., Kreuz C., Chang J.-S., Hwang Y. K., Marsaud V., Bories P.-N., Cynober L., Gil S., Férey G., Couvreur P., Gref R. (2010). Porous Metal–Organic-Framework
Nanoscale Carriers as a Potential Platform for Drug Delivery and Imaging. Nat. Mater..

[7] Li R., Chen T., Pan X. (2021). Metal–Organic-Framework-Based
Materials for Antimicrobial Applications. ACS
Nano.

[8] Bhunia S., Deo K. A., Gaharwar A. K. (2020). 2D Covalent Organic Frameworks for
Biomedical Applications. Adv. Funct. Mater..

[9] Vasquez T. E. J., Saldaña C., Muzikar K. A., Mashek D., Liu J. M. (2016). Searching for Synthetic
Antimicrobial Peptides: An
Experiment for Organic Chemistry Students. J.
Chem. Educ..

[10] Scott C., Wisdom N.-H., Coulter K., Bardin S., Strap J. L., Trevani L. (2023). Interdisciplinary Undergraduate Laboratory
for an Integrated
Chemistry/Biology Program: Synthesis of Silver Nanoparticles (AgNPs)-Cellulose
Composite Materials with Antimicrobial Activity. J. Chem. Educ..

[11] Rodrigues C. A. B., Neto I., Rijo P., Afonso C. A. M. (2018). Synthesizing
a Berberine Derivative and Evaluating Antimicrobial Activity To Reinforce
with Students the Potential Significance of Small Chemical Structure
Changes for Biological Systems. J. Chem. Educ..

[12] Lyle S. J., Flaig R. W., Cordova K. E., Yaghi O. M. (2018). Facilitating Laboratory
Research Experience Using Reticular Chemistry. J. Chem. Educ..

[13] Lisensky G. C., Yaghi O. M. (2022). Visualizing Pore
Packing and Topology in MOFs. J. Chem. Educ..

[14] Todd C., Ceballos C. M., So M. C. (2022). Synthesis, Characterization, and
Evaluation of Metal–Organic Frameworks for Water Decontamination:
An Integrated Experiment. J. Chem. Educ..

[15] Benedetto G., Cleary B. M., Morrell C. T., Durbin C. G., Brinks A. L., Tietjen J., Mirica K. A. (2023). CD-MOF-1 for CO2 Uptake: Remote and
Hybrid Green Chemistry Synthesis of a Framework Material with Environmentally
Conscious Applications. J. Chem. Educ..

[16] Mohammed A. K., Pena-Sánchez P., Pandikassala A., Gaber S., AlKhoori A. A., Skorjanc T., Polychronopoulou K., Kurungot S., Gándara F., Shetty D. (2023). Salicylaldehydate Coordinated Two-Dimensional-Conjugated
Metal–Organic Frameworks. Chem. Commun..

[17] Mohammed A.
K., Gaber S., Raya J., Skorjanc T., Elmerhi N., Stephen S., Sánchez P. P., Gándara F., Hinder S. J., Baker M. A., Polychronopoulou K., Shetty D. (2023). Crystallizing Covalent Organic Frameworks
from Metal
Organic Framework through Chemical Induced-Phase Engineering. Sci. Rep..

[18] Ngece K., Khwaza V., Paca A. M., Aderibigbe B. A. (2025). The Antimicrobial
Efficacy of Copper Complexes: A Review. Antibiotics.

[19] Salah I., Parkin I. P., Allan E. (2021). Copper as
an Antimicrobial Agent:
Recent Advances. RSC Adv..

[20] Zheng Z., Cui J., Wu S., Cao Z., Cao P. (2025). Engineering Metal-Organic
Frameworks for Enhanced Antimicrobial Efficacy: Synthesis Methodologies,
Mechanistic Perspectives, and Versatile Applications. Journal of Functional Biomaterials..

[21] Rauf A., Ahmad Khawaja A., Javed M., Mahmood S., Iqbal S., Nadeem S., Jahangir M., Ahmad M., Bahadur A., Alshalwi M. (2024). Highly Synergistic Antibacterial Activity of Copper
(II)-Based Nano Metal–Organic Framework. Inorg. Chem. Commun..

[22] Li Q.-J., Xing F., Wu W.-T., Zhe M., Zhang W.-Q., Qin L., Huang L.-P., Zhao L.-M., Wang R., Fan M.-H., Zou C.-Y., Duan W.-Q., Li-Ling J., Xie H.-Q. (2025). Multifunctional
Metal-Organic Frameworks as Promising Nanomaterials for Antimicrobial
Strategies. Burn. trauma.

[23] Siwayaprahm P., Sawangphruk M., Bouson S., Krittayavathananon A., Phattharasupakun N. (2017). Antifungal
Activity of Water - Stable Copper-Containing
Metal-Organic Frameworks. R. Soc. Open Sci..

