# Supplementary material for: Antifungal Activity of Conjugated Metal Organic Frameworks: A Multidisciplinary Undergraduate Laboratory Experiment
Source: J Chem Educ. 2026 May 19;103(6):3340–5. doi: 10.1021/acs.jchemed.5c01733 (PMC13262747; doi:10.1021/acs.jchemed.5c01733)
Supplement: Supplementary file 1 [file ed5c01733_si_001.docx]

Antifungal activity of conjugated metal organic frameworks: a multidisciplinary undergraduate laboratory experiment

Tina Skorjanc,^a,^* Safa Gaber,^a^ Martina Bergant Marušič,^c^ Dinesh Shetty,^b,^* Matjaz Valant^a^

*^a^* Materials Research Laboratory, University of Nova Gorica, Vipavska 11c, 5270 Ajdovscina, Slovenia

*^b^* Department of Chemistry and Center for Catalysis & Separations (CeCaS), Khalifa University of Science & Technology, Abu Dhabi, P.O. Box 127788, United Arab Emirates

^c^ Laboratory for Environmental and Life Sciences, University of Nova Gorica, Vipavska 13, 5000 Nova Gorica, Slovenia

[**1.1.** **Objectives** 2](#_Toc215651971)

[**1.2.** **Introduction** 2](#_Toc215651972)

[**1.3.** **Chemistry** 3](#_Toc215651973)

[**1.3.1.** **Experimental procedure** 3](#_Toc215651974)

[**1.3.2.** **Laboratory session (6 hours)** 3](#_Toc215651975)

[**1.3.3.** **Hazards and safety.** 4](#_Toc215651976)

[**1.3.4.** **Ideas for student discussion** 4](#_Toc215651977)

[**1.3.5.** **Additional notes for instructors.** 4](#_Toc215651978)

[**1.3.5.1.** **General Remarks** 4](#_Toc215651979)

[**1.3.5.2.** **List of CAS numbers of reagents used** 5](#_Toc215651980)

[**1.3.5.3.** **Experiment’s photos** 5](#_Toc215651981)

[**1.3.5.4.** **Answers to the ideas for students’ discussion.** 5](#_Toc215651982)

[**1.4.** **Microbiology** 7](#_Toc215651983)

[**1.4.1.** **Experimental procedure** 7](#_Toc215651984)

[**1.4.2.** **Laboratory session (2+2 hours)** 7](#_Toc215651985)

[**1.4.3.** **Hazards and safety.** 9](#_Toc215651986)

[**1.4.4.** **Ideas for students discussion** 9](#_Toc215651987)

[**1.4.5.** **Additional notes for instructors.** 9](#_Toc215651988)

[**1.4.5.1.** **General Remarks** 9](#_Toc215651989)

[**1.4.5.2.** **List of CAS numbers of reagents used** 10](#_Toc215651990)

[**1.4.5.3.** **Answers to the ideas for students’ discussion.** 10](#_Toc215651991)

[**1.4.5.4.** **Instructor notes for the experiments** 10](#_Toc215651992)

[**1.5.** **References** 11](#_Toc215651998)

***1.6. Post-Laboratory Student Evaluation Survey............................................................................****12*

1. **Students’ Handout**
   1. **Objectives**

- Synthesize and purify a metal-organic framework (MOF);
- Understand the mechanism of formation of coordination bonds in a MOF;
- Investigate antifungal activity of the prepared MOF by various methods and understand the differences in the information extracted from each of them;
- Appreciate the multidisciplinary nature of modern science.
  1. **Introduction**

Porous materials, including porous organic polymers (POPs), metal-organic frameworks (MOFs) and covalent organic frameworks (COFs), are emerging classes of materials characterized by extensive surface areas which can extend to several thousand square meters per gram of material. In 2025, the Nobel Prize in Chemistry was awarded for the discovery of MOFs. These are materials composed of metal nodes linked into extended structures by organic linkers, where a coordination bond develops between the metal centers and the organic linkers. Herein, you will use mechanochemistry as a green method of preparing Cu(II)-based MOFs.

The work presented here is divided in two different and independent parts: synthesis of a MOF and a microbiological assay. In the synthesis part, students are asked to prepare, purify, and perform some basic characterizations of a Cu(II)-based MOF. In the microbiology part, different groups of students will take different approaches to evaluating the microbiological, specifically antifungal, activity of the prepared material. Finally, student groups will exchange data and critically evaluate each microbiology approach.


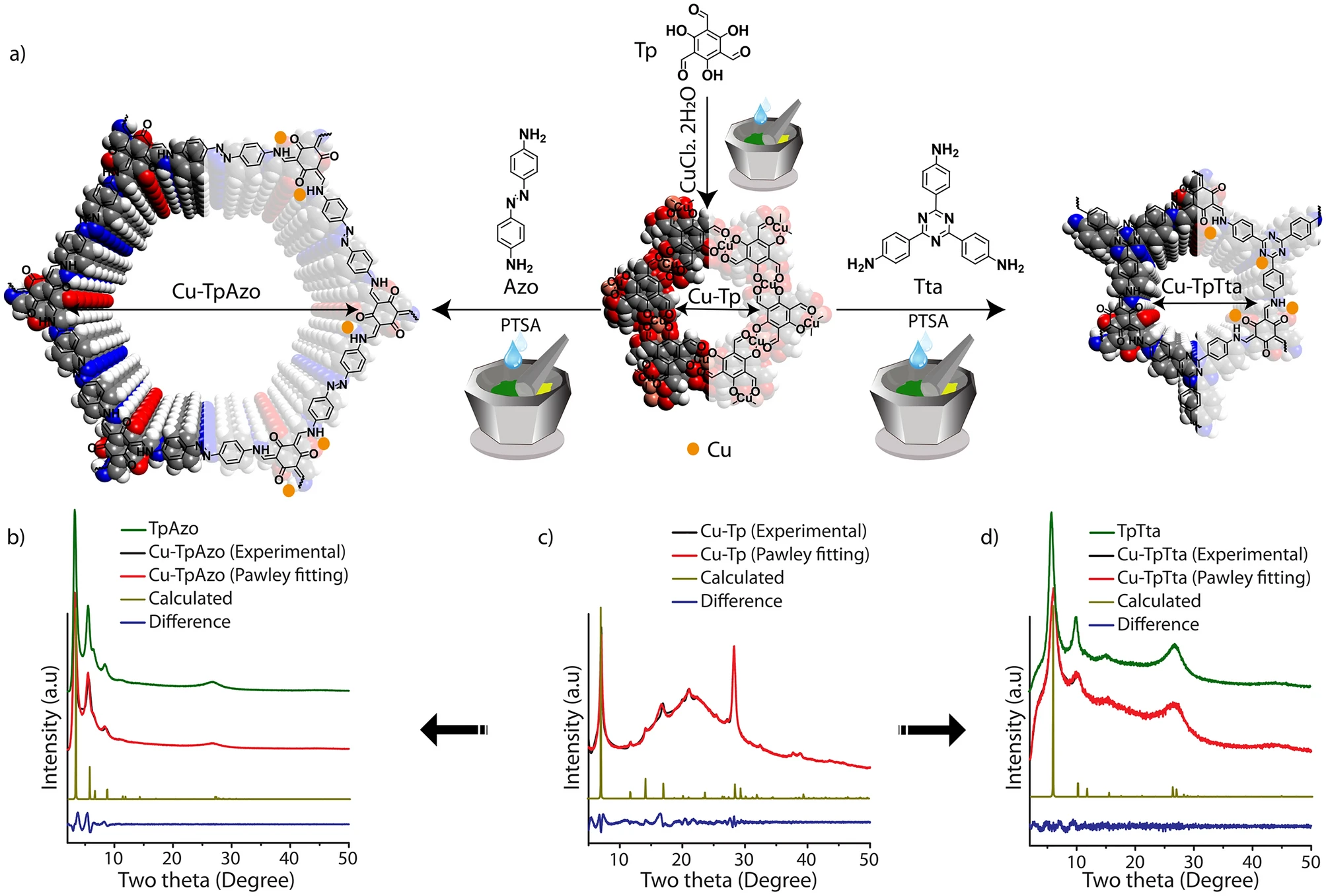


Figure S1: Synthesis of the CuTp MOF. Reproduced with permission from ref. 1.

- 1. **Chemistry**
     1. **Experimental procedure**

| **Required Laboratory Materials** |  |
| --- | --- |
| - Mortar and pestle - Weighing paper - Spatula - P200 pipette - P200 pipette tips - A balance - Gloves | - Labcoat - Safety goggles - Centrifuge tubes - Centrifuge - Oven - Vacuum dryer - Parafilm |
|  |  |
| **Required Reagents** |  |
| - CuCl_2_·2H_2_O - 1,3,5-triformylphloroglucinol (Tp) - Deionized water - N,N’-dimethylacetamide (DMA) | - Tetrahydrofuran (THF) - Acetone |

- - 1. **Laboratory session (6 hours)**

1. Weigh 0.15 mmol (31.5 mg) of the Tp linker and place it into a mortar.
2. Weigh 0.225 mmol (38.4 mg) of CuCl_2_·2H_2_O and add it to a mortar.
3. Using a pestle, mechano-mix the mixture thoroughly until you get a solid paste.
4. Add 50 µL of water to the mixture, so that you can obtain a more uniform mixture.
5. Transfer the mixture into a closed container and place it in the oven pre-heated to 90 °C. Leave the rection mixture in the oven for 4 hours.
6. Remove the solid from the oven and cool it down to room temperature.
7. Transfer the powder into a 15-mL centrifuge tube and add DMA. Shake the tube thoroughly to wash out any impurities soluble in DMA. Centrifuge for 3 minutes at 5000 rpm. Remove and discard the supernatant.
8. Repeat the same washing procedure with fresh DMA. **Note 1.**
9. Repeat the washing procedure with the following solvents: THF, water, acetone.
10. Dry the materials in a vacuum drier at 40 °C overnight.
11. Perform two quick characterizations of the dried samples, namely measure the FT-IR spectrum and collect the PXRD pattern. Check how well they match the reported data.

**Note 1.** Pay attention to the colour of the supernatant. Once you finish washing with every solvent, the supernatant should appear (nearly) transparent.

- - 1. **Hazards and safety.**

**CuCl_2_·2H_2_O** CAS (10125-13-0) Hazardous in case of skin contact (irritant), eye contact (irritant), ingestion, or inhalation. Very toxic to aquatic life with long-lasting effects.

**1,3,5-triformylphloroglucinol** CAS (22363-11-7) Hazardous in case of skin contact (irritant), eye contact (irritant), ingestion, or inhalation.

**N,N’-dimethylacetamide** CAS (127-19-5) Hazardous in case of skin contact (irritant), eye contact (serious irritant), ingestion, or inhalation. May damage fertility or the unborn child. May cause damage to organs through prolonged or repeated exposure.

**Tetrahydrofuran** CAS (109-99-9) Highly flammable liquid and vapor. Hazardous in case of skin contact (irritant), eye contact (irritant), or inhalation (irritant). May cause drowsiness or dizziness.

**Acetone** CAS (67-64-1) Highly flammable liquid and vapor. Hazardous in case of eye contact (serious irritant). May cause drowsiness or dizziness. Repeated exposure may cause skin dryness or cracking.

**Use protective clothing, gloves, safety goggles and perform all the manipulations in the fume hood.**

- - 1. **Ideas for student discussion**

1. **Propose a reaction mechanism. Compare your understanding with reported information.**
2. **Explain the roles of mechanical grinding and heating in the formation of the CuTp MOF.**
3. **What information can you extract from each of the microbiological experiments? Do results from one assay match the results from another? Why is it useful to use three different methods?**
4. **Can you find alternative methods for studying antifungal activity of a MOF?**
   - 1. **Additional notes for instructors.**
        1. **General Remarks**

All reagents and solvents used are commercially available in Aldrich, BLDPharm or Merck and have been used without further purification. Fourier transform infrared (FT-IR) spectra were obtained using a Bruker Invenio-R spectrometer with a universal A225/QHP Platinum-ATR (attenuated total reflection) accessory in the 400-4000 cm^–1^ region. PXRD patterns were recorded on Rigaku SmartLab SE with a Cu Kα source, a step size of 0.03° and incident slit of 0.1 mm.

- - - 1. **List of CAS numbers of reagents used**

**CuCl_2_·2H_2_O** CAS (10125-13-0)

**1,3,5-triformylphloroglucinol** CAS (22363-11-7)

**N,N’-dimethylacetamide** CAS (127-19-5)

**Tetrahydrofuran** CAS (109-99-9)

**Acetone** CAS (67-64-1)

- - - 1. **Experiment’s photos**

| 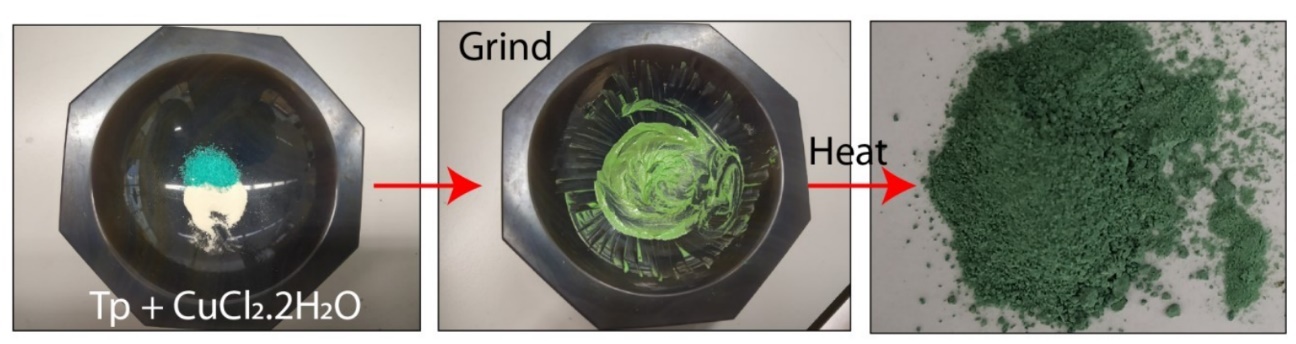 |  |
| --- | --- |

Figure S2: Photographs of different stages of mechanochemical synthesis of CuTp. Note the color changes. Reproduced with permission from ref. 2.

- - - 1. **Answers to the ideas for students’ discussion.**

1. **Propose a reaction mechanism. The students can compare their rationalization with reported information.**

This is a Lewis acid-base reaction, where the metal centers (Cu^2+^) serve as Lewis acids and the aldehyde and hydroxyl groups of Tp act as Lewis bases. Cu^2+^ is able to form four coordination bonds which results in every metal ion being shared among two neighboring Tp molecules.

1. **Explain the roles of mechanical grinding and heating in the formation of this MOF.**

Mechanical grinding ensures the formation of a uniform reaction mixture, whereas heating at 90 °C ensures sufficient thermal energy to overcome the activation energy of the reacture and form a MOF structure.

1. **What information can you extract from each of the microbiological experiments? Do results from one assay match the results from another? Why is it useful to use three different methods?**

It is useful to test the antifungal activity by different means because every experiment gives us a different piece of information. A simple measurement of O.D. measures turbidity and gives an indication of the **number of cells**; the higher the O.D., the more cells there are in the sample. However, if the tested compound absorbs light at 600 nm, this can interfere with the results. This method does not indicate whether the cells are dead or alive. In contrast, an assay with Presto Blue measures the **metabolic activity** of cells and relies on a resazurin-based reagent which is reduced by metabolically active cells into a fluorescent product called resorufin. This method will therefore give an indication of metabolically active cells, which may not be all the cells in the sample. Finally, an assay with Crystal Violet is used to study the **biofilm** biomass. Biofilms are typically more difficult to eradicate than planktonic bacteria and fungi. Crystal Violet binds to negatively charged molecules in fungal cells and extracellular polymeric substances (EPS) in biofilms. If the tested MOF is effectively inhibiting biofilm formation, less dye will bind.

1. **Can you find alternative methods for studying antifungal activity of a MOF?**

Disk diffusion method could be an alternative. It would require the preparation of agar plates and a MOF pressed into a tablet. This method would require students to measure the diameter of the zone of inhibition around the tablet.

Plating aliquots of fungi suspensions on agar plates and counting the number of colonies that form the next day could also be used, but this is considered a low throughput and time-consuming method.

- 1. **Microbiology**
     1. **Experimental procedure**

| **Required Laboratory Materials** |  |
| --- | --- |
| - Bunsen burner or access to a biosafety cabinet - Sterile loops (wire/plastic) - Sterile 48-well plates - Marker pen - Sonicator | - Pasteur pipettes (plastic/glass) - Incubator - Microplate reader (absorbance, fluorescence) - UV-Vis spectrophotometer - Pipettes and pipette tips |
|  |  |
| **Required Reagents** |  |
| - Ethanol 70% - Sterile water - CuTp MOF prepared by students - Phosphate buffered saline (PBS) - Glucose | - Dimethyl sulfoxide (DMSO) - Presto Blue Reagent - Crystal Violet - Acetic acid - Yeast-peptone-dextrose (YPD) broth |
|  |  |
| **Microorganisms** |  |
| - *Candida albicans* ATCC 10231 |  |

- - 1. **Laboratory session (2+2 hours)**

1. Work in aseptic conditions. You will receive a stock of *C. albincans* suspension from your instructor with a given O.D. value. Dilute the suspension so that you get 5 mL of suspension with O.D. = 0.1.

$$V_{initial}=\frac{V_{final} \cdot{O.D.}_{final}}{{O.D.}_{initial}}$$

1. In aseptic conditions, further dilute the culture at O.D.= 0.1 **1:50** in fresh YPD media to get a working solution.
2. Prepare 6 serial dilutions of the material to be tested in 5% DMSO - 95% YPD broth mixture (highest final concentration in the well 1 mg/mL, 100 µL per well) into the wells of a sterile 48-well plate. Use a sonicator to disperse the particles well. Work in triplicates. **Note 1.**
3. Pipette 150 µl of the fungal cells (working solution) onto each dilution of the material and incubate in an incubator without shaking at 37°C overnight.
4. Do not forget to include a control, where the MOF sample is replaced by the YPD medium.

The following day, each group follows one of the following protocols.

**Optical density measurements:**

6A. Using a microplate reader, set up a method that measures the absorbance at 600 nm.

7A. Measure the absorbance in every well. Calculate the average of the three triplicates for every condition and divide with the average of the controls.

$$\% yeast growth= \frac{Absorbance @ test}{Absorbance @ control}\cdot100 \%$$

8A. Make a plot of the % yeast growth as a function of MOF concentration.

**Presto Blue reagent measurements:**

6B. Add 5 µL of the Presto Blue reagent to every well and incubate the plate in the dark at 37 °C for 30 minutes.

7B. Using a microplate reader, set up a method that measures the fluorescence intensity at 590 nm and uses an excitation wavelength of 560 nm.

8B. Read the fluorescence intensity in every well. Calculate the average of the three triplicates for every condition and divide with the average of the controls.

$$\% viability= \frac{Fluorescence @ test}{Fluorescence @ control}\cdot100$$

9B. Make a plot of the % viability as a function of MOF concentration.

**Crystal Violet measurements:**

6C. Remove the planktonic cells from the wells and wash every well with 200 µL of sterile PBS.

7C. Stain the biofilms with 0.2 % solution of Crystal Violet prepared in water for 30 minutes. Incubation should be done in the dark.

8C. Remove excess Crystal Violet with a pipette and wash every well with PBS three times. Leave the cells to dry on air.

9C. Destain the biofilms with 33 % acetic acid for 15 minutes.

10C. Measure the O.D. at 595 nm using a microplate reader.

11C. Calculate the percent biofilm inhibition using the following formula:

$$\% biofilm inhibition= \frac{(O.D. @ control-O.D. @ test)}{(O.D. @ control-O.D. @ blank)}\cdot100$$

9B. Make a plot of the % biofilm inhibition as a function of MOF concentration.

**Note 1:** If you are conducting the Crystal Violet experiment, add 1 % of glucose to the YPD medium for better attachment of cells to the well.^3^

- - 1. **Hazards and safety.**

**Ethanol** CAS (64-17-5) Hazardous in case of skin contact (irritant and permeator), eye contact (irritant), inhalation, or ingestion.

**Dimethyl sulfoxide** CAS (67-68-5) Irritating to the skin, eyes, nose, throat and respiratory tract.

**CuCl_2_·2H_2_O** CAS (10125-13-0) Hazardous in case of skin contact (irritant), eye contact (irritant), ingestion, or inhalation. Very toxic to aquatic life with long-lasting effects.

**1,3,5-triformylphloroglucinol** CAS (22363-11-7) Hazardous in case of skin contact (irritant), eye contact (irritant), ingestion, or inhalation.

**Crystal Violet** CAS (548-62-9) Hazardous in case of skin contact (irritant), eye contact (irritant), ingestion, or inhalation. Very toxic to aquatic life with long-lasting effects. Suspected of causing genetic defects (mutagenic).

***Candida albicans* (ATCC 10231)**. A **Biosafety Level 1 (BSL-1)** yeast was used to evaluate antimicrobial activity. BSL-1 denotes the most basic level of laboratory containment, requiring only standard microbiological practices without the need for specialized equipment or barriers—aside from a hand-washing sink. Alongside general safety measures such as wearing goggles, gloves, and lab coats, students conduct their work near open flames from Bunsen burners or inside the biosafety cabinets. All liquid materials that come into contact with *C. albicans* should be disinfected in 1% bleach prior to disposing in the sink. Other, non-liquid materials that are in contact with fungi should be autoclaved and disposed of as regular mixed waste.

**Use protective clothing, gloves, safety goggles and perform all the manipulations in the fume-hood.**

- - 1. **Ideas for students discussion**

1. **Compare the antimicrobial activity measured by the three different methods. Which is most/least suitable for MOFs?**
2. **What results in antimicrobial assays would you expect to obtain with metal salt and organic linker, i.e. the building blocks of the MOF?**
   - 1. **Additional notes for instructors.**
        1. **General Remarks**

*Candida albicans* ATCC 10231 strain was used in these microbiological experiments. A single colony should be inoculated in 5 mL YPD broth overnight (37 °C, with shaking) and the obtained suspension should be given to the students the next day. The O.D. of this suspension should be measured before it is given to students.

- - - 1. **List of CAS numbers of reagents used**

**Ethanol** CAS (64-17-5)

**Dimethylsulfoxide** CAS (67-68-5)

**CuCl_2_·2H_2_O** CAS (10125-13-0)

**1,3,5-triformylphloroglucinol** CAS (22363-11-7)

**Crystal Violet** CAS (548-62-9)

- - - 1. **Answers to the ideas for students’ discussion.**

1. **Compare the antimicrobial activity measured by the three different methods. Which is most/least suitable for MOFs?**

If the chosen MOF absorbs light around 600 nm, then the O.D. measurements may be least suitable for the evaluation of its antimicrobial activity. Higher concentrations of the MOF will contribute more significantly to the O.D. reading, thereby counteracting the potential reduction in *C. albicans* proliferation. The Presto Blue assay measures the metabolic activity of the cells, whereas the Crystal Violet assay measures the inhibition of biofilm formation. These two assays therefore give entirely different pieces of information, which complement each other.

1. **What results in antimicrobial assays would you expect to obtain with metal salt and organic linker, i.e. the building blocks of the MOF?**

Generally, heavy metals exhibit higher toxicity than simple organic molecules, but this highly depends on the concentration, type of metal, and type of organic linker. If the same experiment were to be repeated with the proportion of metal ions present in the MOF, the effect of free metal would likely be more toxic. This is because in the 24-hour time frame, the MOF is unlikely to degrade and may not enter the cells due to the particle size. In contrast, metal salts can enter the cells more easily and can interfere with various metabolic pathways, thus bringing about a faster inhibition. However, MOFs in this context have various other advantages: their pores can be loaded with various drugs for combination therapies, they can hydrolyze in presence of a stimulus (e.g. acidic pH), which ensures enhanced toxicity in the desired conditions compared to normal conditions, and they can exhibit sustained toxicity for longer time periods, thus limiting the side effects.

- - - 1. **Instructor notes for the experiments**

1. For practical reasons, the initial *C. albicans* suspensions should be prepared in advance, specifically the day before the experiment.
2. The experiment in the microbiology laboratory can be completed in about 2 hours with an additional 2 hours the next day. O.D. measurements are quicker to perform than the Presto Blue assay. The Crystal Violet assay takes the most time, but not more than 1.5 hours.
3. It is recommended that the instructor sets up the method for the absorbance and/or fluorescence measurements prior to the student session.

1. 5. **References**

(1) Mohammed, A. K.; Gaber, S.; Raya, J.; Skorjanc, T.; Elmerhi, N.; Stephen, S.; Sánchez, P. P.; Gándara, F.; Hinder, S. J.; Baker, M. A.; Polychronopoulou, K.; Shetty, D. Crystallizing Covalent Organic Frameworks from Metal Organic Framework through Chemical Induced-Phase Engineering. *Sci. Rep.* **2023**, *13* (1), 19443.

(2) Mohammed, A. K.; Pena-Sánchez, P.; Pandikassala, A.; Gaber, S.; AlKhoori, A. A.; Skorjanc, T.; Polychronopoulou, K.; Kurungot, S.; Gándara, F.; Shetty, D. Salicylaldehydate Coordinated Two-Dimensional-Conjugated Metal–Organic Frameworks. *Chem. Commun.* **2023**, *59* (18), 2608–2611.

(3) David, H.; Nithya, K.; Shankar Salian, L.; Dandela, R.; Suresh, D.; Amali, A. J.; Solomon, A. P. Exploring the Potential of Covalent Organic Frameworks to Combat Candida Albicans Biofilm Formation and Persistence. *ACS Appl. Bio Mater.* **2025**, *8* (9), 7715–7727.

**Post-Laboratory Student Evaluation Survey: Antifungal Activity of Conjugated Metal–Organic Frameworks**

**Section 1: General Satisfaction**

On a scale of 1 to 5, please evaluate each of the following aspects of the experiment.

|  | Strongly disagree | Disagree | Neutral | Agree | Strongly agree |
| --- | --- | --- | --- | --- | --- |
| The laboratory experiment was well organized. | 1 | 2 | 3 | 4 | 5 |
| The instructions provided were clear and easy to follow. | 1 | 2 | 3 | 4 | 5 |
| The workload was appropriate for the time available. | 1 | 2 | 3 | 4 | 5 |
| I felt adequately prepared to perform the experiment. | 1 | 2 | 3 | 4 | 5 |
| The experiment was engaging and interesting. | 1 | 2 | 3 | 4 | 5 |
| Overall, I am satisfied with this laboratory experience. | 1 | 2 | 3 | 4 | 5 |

**Section 2: Learning Objectives**

On a scale of 1 to 5, please evaluate to what extent were each of the following learning objectives met.

|  | Not achieved | Barely achieved | Partly achieved | Mostly achieved | Fully achieved |
| --- | --- | --- | --- | --- | --- |
| I can perform mechanochemical synthesis and purification of a MOF. | 1 | 2 | 3 | 4 | 5 |
| I can weigh chemicals, grind, thermally treat, and wash them. | 1 | 2 | 3 | 4 | 5 |
| I can properly document procedures and results (yield, observations). | 1 | 2 | 3 | 4 | 5 |
| I can prepare samples for FT-IR and PXRD measurements. | 1 | 2 | 3 | 4 | 5 |
| I can compare obtained spectra with reference data. | 1 | 2 | 3 | 4 | 5 |
| I can correctly assign FT-IR peaks and identify PXRD reflections. | 1 | 2 | 3 | 4 | 5 |
| I can prepare *C. albicans* suspensions and perform serial dilutions. | 1 | 2 | 3 | 4 | 5 |
| I can work under sterile conditions and set up microplate experiments. | 1 | 2 | 3 | 4 | 5 |
| I understand how to interpret fungal growth results. | 1 | 2 | 3 | 4 | 5 |
| I can perform optical density, Presto Blue, and Crystal Violet assays. | 1 | 2 | 3 | 4 | 5 |
| I understand the purpose and differences between the assays. | 1 | 2 | 3 | 4 | 5 |
| I can compare results across different groups. | 1 | 2 | 3 | 4 | 5 |
| I can critically evaluate assay limitations (e.g., MOF absorbance effects). | 1 | 2 | 3 | 4 | 5 |
| I contributed effectively to group work. | 1 | 2 | 3 | 4 | 5 |
| I improved my ability to interpret shared data and solve problems | 1 | 2 | 3 | 4 | 5 |

**Section 3: Open-ended questions**

1. What was the most valuable part of the experiment?
2. What aspect of the experiment was the most challenging?
3. Do you have any suggestions for improving the experiment?

**Post-Laboratory Student Evaluation Survey: Survey Results (n = 7)**

**Section 1: General Satisfaction**

|  | Average score |
| --- | --- |
| The laboratory experiment was well organized. | 4.7 |
| The instructions provided were clear and easy to follow. | 4.3 |
| The workload was appropriate for the time available. | 4.5 |
| I felt adequately prepared to perform the experiment. | 4.2 |
| The experiment was engaging and interesting. | 4.7 |
| Overall, I am satisfied with this laboratory experience. | 4.7 |

**Section 2: Learning Objectives**

|  | Average score |
| --- | --- |
| I can perform mechanochemical synthesis and purification of a MOF. | 4.7 |
| I can weigh chemicals, grind, thermally treat, and wash them. | 5 |
| I can properly document procedures and results (yield, observations). | 4.8 |
| I can prepare samples for FT-IR and PXRD measurements. | 4.5 |
| I can compare obtained spectra with reference data. | 4 |
| I can correctly assign FT-IR peaks and identify PXRD reflections. | 3.7 |
| I can prepare *C. albicans* suspensions and perform serial dilutions. | 4.8 |
| I can work under sterile conditions and set up microplate experiments. | 4.7 |
| I understand how to interpret fungal growth results. | 4.8 |
| I can perform optical density, Presto Blue, and Crystal Violet assays. | 4.7 |
| I understand the purpose and differences between the assays. | 4.7 |
| I can compare results across different groups. | 4.8 |
| I can critically evaluate assay limitations (e.g., MOF absorbance effects). | 4.8 |
| I contributed effectively to group work. | 4.7 |
| I improved my ability to interpret shared data and solve problems | 4.8 |
